# Dietary Polyphenol Intake Associated with Adiposity Indices among Adults from Low to Medium Socioeconomic Status in a Suburban Area of Kuala Lumpur: A Preliminary Findings

**DOI:** 10.21315/mjms2019.26.6.7

**Published:** 2019-12-30

**Authors:** Hanisah Rosli, Yifang Kee, Suzana Shahar

**Affiliations:** 1 Dietetics Program, Faculty of Health Sciences, Universiti Kebangsaan Malaysia, Kuala Lumpur, Malaysia; 2 Faculty of Allied Health Sciences, Cyberjaya University College of Medical Sciences, Cyberjaya, Selangor, Malaysia; 3 Nutritional Science Program, Faculty of Health Sciences, Universiti Kebangsaan Malaysia, Kuala Lumpur, Malaysia

**Keywords:** polyphenol, food intake, adults, obesity, Malaysia

## Abstract

**Background:**

Researches on polyphenols have been the interest of few parties due to its possible roles in the prevention of obesity. However, studies regarding this topic are still limited. Therefore, this study was conducted to examine the relationship between the intake of polyphenols with adiposity indices among middle-aged adults.

**Methods:**

This cross-sectional study involved 227 adults aged 40 to 59 years at low-cost housing flats in suburban area of Cheras, Kuala Lumpur. Data collection involved food frequency questionnaire (FFQ) for polyphenols and international physical activity questionnaire (IPAQ). Subjects were measured for anthropometric parameters including height, weight, waist and neck circumferences (NC), and body fat percentage. The polyphenol intake from the diet was estimated using local polyphenol database built according to PHENOL-EXPLORER.

**Results:**

The average intake of polyphenol of subjects was 1815 (672) mg/day. The main food sources of polyphenol were coffee with milk, followed by chocolate milk and red beans. A higher polyphenol intake according to quartile was significantly associated with a lower neck circumference (*χ*^2^ = 8.30, *P* = 0.040), waist circumference (*χ*^2^ = 8.45, *P* = 0.038) and body fat percentage (*χ*^2^ = 8.06, *P* = 0.045). Binomial logistic regression analysis showed that the association remained significant for the neck circumference (*P* = 0.032), after controlling for age, household income, energy intake and physical activity level. More subjects with normal NC had higher intake of polyphenols (50th percentile and above). In contrast, subjects with high NC showed lower percentiles of polyphenols intake (50th percentile and below).

**Conclusion:**

The result showed that polyphenol intake was associated with neck circumference and thus it can be suggested that polyphenol intake is associated with obesity.

## Introduction

For many years, the prevalence of obesity has been rising at an alarming rate and has become a major worldwide health problem. Many interventions have been introduced to solve this problem, however some of it failed to show any positive results in reducing obesity. Recently, researchers, consumers and manufacturers have shown interest in natural products especially polyphenols due to their role in preventing many diseases and possible potential to reduce obesity and problems related to adiposity. Several researches have shown that the addition of foods containing polyphenols in the diet together with healthy eating habit and exercise may help to prevent obesity and to maintain an ideal body weight (1). Besides acting as a powerful antioxidant, polyphenols may suppress the adipose tissue, stimulate lipolysis, inhibit preadipocytes differentiation and thus reduce the adipose tissue mass (2). Many cell culture and animal studies suggested that intake of foods containing certain polyphenols altered lipid and energy metabolism and may help in weight loss and prevent weight gain (1). Numerous bioactive components of polyphenols have been indicated to be potentially useful in obesity treatment, including catechins from green and white teas (3), fruits such as blueberries with anthocyanin (4), red grapes and wine with resveratrol (5) and curcumin from spice like turmeric (6).

Overweight and obesity is a condition which affects all age groups. However, in particular, middle-aged adults aged 45 to 59 years are at higher risk for gaining weight (7). This may be due to changes in biological functions and work and family commitments. Middle-aged adults who are overweight and obese have higher risk of developing various illnesses during old age and have shorter life expectancy (7).

Although there were several cell cultures and animal studies explored the impact of polyphenol on adiposity, the human consumption studies are still limited (1). Most of the studies examined the polyphenolic content of foods without relating to the human body function and health outcomes. Hence this study aims to examine the relationship between dietary polyphenol intake and adiposity indices as well as to determine the consumption pattern and important food sources of polyphenol among sub-urban middle-aged adults in Kuala Lumpur.

## Subjects and Methods

This is a cross sectional study with enrolment of 227 middle-aged adults (aged 40 – 59 years) to determine the association between dietary polyphenol intake and adiposity. Middle-aged adults were selected since this study is interested to determine the trend of polyphenols intake and its association with adiposity indices prior to reaching elderly age. Middle-aged adults may be at risk of developing non-communicable disease, which are associated with adiposity and dietary intake, due to their increasing age and lifestyles. Furthermore, adiposity indices and polyphenols intake may be affected by poor appetite and changes in metabolism which occur during old age, and this will affect the result of this study.

Subjects were recruited from housing flats scheme targeted for low to middle income group in suburban area of Cheras, Kuala Lumpur. This area was selected since subsequent clinical trial after this preliminary study would be conducted in a public hospital near the area. Additionally, researchers were attached to the same hospital. This would facilitate both subjects and researchers in conducting subsequent study.

Sample size was calculated by using the formula by Cochran (8) with 8% level of accuracy of the mean with 95% certainty. Mean of polyphenols intake of 2770.7 (1552.4) mg/day were taken from a previous study by (9). After taking into consideration 20% dropout rate, the sample size calculated was 227.

Convenient sampling was used in this study with pamphlets were given to all housing flats with occupants aged 45 to 59 years. The details of the subjects were given by a local city hall, who was responsible in management and maintenance of the housing flats. Data were collected at nearby community halls. Subjects were excluded from the research if they had any chronic diseases or physical disability. Additionally, pregnant or lactating subjects and those who were on hormone-replacement subjects were also excluded in this study. Subjects who were taking food supplements or had changes in their dietary habits for the past three months were excluded from this study as well.

Subjects were interviewed by researchers with nutrition background, using a structural questionnaire that included information on sociodemographic and medical history. Additionally, subjects were also interviewed about their polyphenols intakes. Adiposity indices measured included percentage of body fat, neck and waist circumferences and body mass index (BMI) (10, 11). Prior to the data collection, researchers were given intensive training on the research protocol and data collection methods. Subjects were explained about the research and data collection was only continued after the subjects agreed and signed the consent forms.

### Polyphenol Assessment

A validated food frequency questionnaire (FFQ) for polyphenol (9) was used to estimate the intake of dietary polyphenols. The FFQ consists of 117 food items from 9 food categories that are normally consumed by local population. This includes fruits, vegetables, cereals and nuts, caffeinated beverages, juices, chocolate and spices. Subjects were asked in detail about the amount and frequency of intake per day, per week or per month. In order to get an estimation of the amount of intake, household tool measurements such as cups and tablespoons were used. The amount was then converted to weight in grams for polyphenols analysis. FFQ for polyphenols has been validated in a previous study by Suzana et al. (9) against 2-day diet records and diet history questionnaire. Spearman’s rho and Kendall’s Tau-b analysis indicated that there was a significant positive correlation between polyphenol intake estimated from FFQ and reference method (*r* = 0.41, *P* < 0.001; *r* = 0.28, *P* < 0.001). For Bland-Altman plot, 95.7% of scattered plot fell within ± 1.96 SD limits of agreement revealed that there was good agreement between the two methods used (9).

The polyphenol intake was calculated by multiplying the reported consumption frequency, consumed quantity on the FFQ and assigned polyphenol content of each food based on the PHENOL EXPLORER and meta-analysis literatures (9). PHENOL EXPLORER is an online comprehensive database on polyphenols content in food (12). It consisted of polyphenols content in various food from more than 37,000 original data points collected from 638 scientific articles published in peer-reviewed journals.

### Anthropometric Measurements

Body weight and height were measured using TANITA Digital Scale Model HD30 and SECA Bodymeter 208, respectively, using standard methods (13). Neck circumference was measured in the midway of the neck, between midcervical spine and midanterior neck plastic tape (Lufkin Executive Thinline Model W606PM). In men with laryngeal prominence (Adam’s apple), measurement was taken below the prominence (14). Values of ≥ 38 cm for men and ≥ 35 cm for women were considered as high (14). Waist circumference was measured at the midpoint between the lower margin of the last palpable rib and the top of the iliac crest. Values of ≥ 90 cm and ≥ 80 cm for women were considered as high (15). Body fat was assessed by bioelectrical impedence analysis (BIA) using Omron Body Fat Monitor HBF-306. Values of ≥ 25% for men and ≥ 35% for women were considered high (16). Body mass index (BMI) was classified using the recommendations by the World Health Organization (16).

### Physical Activity Assessment

International Physical Activity Questionnaires (IPAQ) was used to assess the subjects’ physical activity level (17). They were asked the duration and number of days that they performed physical activity over the past seven days. Physical activity levels were categorised into three groups, namely, vigorous activity, moderate activity and walking activity (17).

IPAQ has been validated against seven-day physical activity log (PA-log) among the Malaysian community (18). Interclass correlation coefficients showed moderate to good correlations (ICC = 0.54–0.92; *P* < 0.001) on items categorised by intensities and domains. Additionally, κ value of 0.73 was obtained when analysed for total activity. The result from validity analysis showed a significant result across intensities and domains (ρ = 0.67–0.98) (*P* < 0.01). This study demonstrated that IPAQ has good reliability and validity in assessing physical activity level among Malaysians (18).

## Statistical Analysis

SPSS version 20.0 was used to analyse the data. Normality test Kolmogorov-Smirnov test was carried out before any statistical analysis. Categorical variables such as sociodemographic data and adiposity indices were presented as frequency and percentage (%). Polyphenols intake and anthropometric profiles were presented in mean and standard deviation. Pearson’s chi-squared (*χ*^2^) test was used to test the association between polyphenol intake percentile and adiposity indices [body mass index (BMI), neck circumference (NC), waist circumference (WC), body fat percentage]. Subsequently, binomial logistic regression analysis was used to assess the association between dietary polyphenol intake and adiposity indices with adjustment of age, income level, caloric intake and physical activity level. Binomial logistic regression was conducted since the researchers were interested to study the relationship of different percentiles intake of polyphenols with various classifications of adiposity indices with adjustment for cofounding factors. By studying the different percentiles intake of polyphenols, the relationship can be observed for subjects who were categorised as having poor, moderate and high intake of polyphenols. Prior to conducting all analysis, all statistical assumptions were assured not to be violated.

## Results

A total of 227 subjects (38.8% men and 61.2% women) participated in the study, with most of the subjects aged between 50 and 59 years (63.4%) (Table 1). Ethnic distribution of the subjects was 39.5% Malays, 37.0% Chinese and 23.3% Indians. Most of the subjects had education at secondary school level (58.6%), still working (48.5%) and had a moderate household income of between RM1,501 to RM3,500 per month (71.4%). For physical activity level, about half of the women (56.1%) and men (46.6%) subjects had low to moderate physical activity.

This study showed that men subjects had lower BMI (*P* < 0.050) and body fat percentage (*P* < 0.010) as compared to their female counterparts (Table 2). In addition, men also had greater NC than the female subjects (*P* < 0.010) (Table 2).

Additionally, most of the subjects were overweight (40.9% for men and 36.0% for women) (Table 3). In terms of other adiposity indices, more men (72.7%) were categorised as having high NC as compared to women (52.5%). In contrast, more women (81.3%) were identified as having high WC as compared to men (52.3%). For body fat percentage, more men (75.0%) were classified as having high body fat percentage as compared to women (61.9%).

The mean total polyphenol intake of the subjects was 1815 (672) mg/day (Table 4). Polyphenol intake was also calculated according to different food groups (fruits, vegetables, cereals and nuts, juice, caffeinated beverages, soy, chocolate, alcoholic beverages and others). Caffeinated beverages were the main polyphenol provider with contribution of 855.8 (477.8) mg/day, followed by vegetables [298.2 (117.5) mg/day], fruits [221.8 (162.8) mg/day] and cereals and nuts (215.4 (408.1) mg/day]. Other food groups which mostly consists of herbs and spices also provided 156.3 (171.7) mg/day polyphenols. Food groups such as soy [35.0 (57.7) mg/day], juice [31.7 (71.2) mg/day], chocolate [0.3 (2.3) mg/day] and alcoholic beverage [0.1 (1.2) mg/day] contributed polyphenol in small quantity.

The main food contributors in each food group are shown in Table 4. Out of 117 food items, the main food frequently consumed was coffee with milk, which contributed about 20% to the total polyphenol intake, followed by chocolate milk (10%), red beans (7%), black coffee (7%), tea (5%) and others. Most of the polyphenol contributors were from caffeinated beverages. Polyphenols were widely distributed over several food sources, for example fruits, vegetables and others. For other food groups, the main contributors were identified as red beans (61%) from the cereals and nuts group and coffee (43%) from the caffeinated beverages group.

Table 5 shows the relationship between percentile intake of polyphenol and adiposity indices. Results shows that there was a significant relationship between the percentiles of polyphenol intake and NC, WC and body fat percentage (*P* < 0.050). After adjustment of age, income level, caloric intake and physical activity level, the relationship remains significant for NC. More subjects with normal NC had higher intake of polyphenols (50th percentile and above). In contrast, subjects with high NC showed lower percentiles of polyphenols intake (50th percentile and below).

## Discussion

This study shows that men had a higher NC as compared to women (*P* < 0.001). In addition, a total of 72.7% men were categorised as having high NC as compared to women (52.5%). NC has been used as an index for upper-body subcutaneous adipose tissue distribution (14). Additionally, researches have been done to study its relationship with cardiovascular risk factors, insulin resistance, and biochemical components of metabolic syndrome (19–22). Similar with the study by (14) among Chinese adults, men had a 3 cm wider NC than women (*P* < 0.001).

In contrast, this study showed that more women (81.3%) were classified as having high WC as compared to men (52.3%). WC indicates abdominal obesity among the subjects. Similar with the result by the Malaysian National Health and Morbidity Survey (NHMS) 2015 (23), women (60.2%) were found to have a significantly higher prevalence of abdominal obesity than men (38.2%). It can be seen that the prevalence of abdominal obesity in the present study is higher than the national prevalence as stated in (23). This may be due to the location of the data collection. This study concentrated on the urban area as compared to (23), which was conducted in wider geographical areas. The (23) stated that the prevalence of abdominal obesity is higher in urban area as compared to rural areas.

The present study found that the total polyphenol intake was higher than the intake by other studies (24, 25). The explanation for this can partly contributed by several factors. The various dietary habit and food culture may result in different food intake and thus amount of polyphenol intake. The estimation of polyphenol intake in this study was based on the food usually consumed by the local population. Hence, the polyphenol intake might be different from the study of the Western countries. Furthermore, in this study, FFQ was used to assess the polyphenol intake while other studies used diet recall (24, 25). FFQ tend to report higher estimation of intake compared to diet recall and thus give a higher mean polyphenol intake.

In this study, the intake of polyphenol from caffeinated beverage was the highest. The main food contributors from fruits and vegetables in this study were orange, apple and onion. Coffee contributed around half of the polyphenol intake from caffeinated beverage and become the main food source out of the 117 food items. The result is supported by many studies (26–28). Other main contributor for example tea, which contributed around 7% to total polyphenol intake also has been reported as one of the important food sources in many studies (29).

Several studies have investigated the relationship between the polyphenol intake and adiposity indices, however, these studies only focused on specific classes of polyphenol with anthropometric status, such as flavonoid intake and BMI (30). The present study found that there was a significant relationship between the percentile intake of polyphenol intake and adiposity indices but not in the BMI.

Subsequently, analysis was conducted by adjusting of cofounding factors, namely, age, household income, energy intake and physical activity. The result shows a significant relationship of NC and polyphenols intake. More subjects with normal NC were found to have intakes of polyphenols 50th percentile and above. In contrast, subjects with high NC had lower intakes of polyphenols (50th percentile and below). To the best of our knowledge, there is no previous studies examined the relationship between total polyphenol intake and NC. Previous researches have shown that NC has been recognised as a new measure of central obesity (31) and thus it can be suggested that the association of low percentile of polyphenol intake with high NC may be associated with a high central obesity problem. Various researches have shown that polyphenols may potentially affect adiposity and obesity through several mechanisms. Cellular studies demonstrated that dietary polyphenols is responsible for adiposity and obesity reduction by suppressing viability of adipocytes and proliferation of preadipocytes, reducing adipocyte differentiation and triglyceride accumulation, stimulate lipolysis and fatty acid β-oxidation and reduce inflammation (24). With regard to resveratrol, one of polyphenols classes, its anti-obesity effect is contributed by inhibiting preadipocyte differentiation, decreasing adipocyte proliferation, inducing adipocyte apoptosis, decreasing lipogenesis, and promoting lipolysis and fatty acid β-oxidation (24).

The study found that there was no significant relationship between polyphenol intake with BMI. This may probably due to the few reasons. We suggested that the reduction effect of polyphenol on BMI was modulated by certain specific class of polyphenol compared to total polyphenols intake and hence the relationship was neglected or may not exist with the total polyphenol intake and BMI.

## Conclusion

The result of this study elucidates the potentials of polyphenols in the prevention of obesity. More subjects with normal NC were found to have higher intakes of polyphenols. In contrast, subjects with high NC had lower intakes of polyphenols. For future studies, the anti-obesogenic dosage of polyphenols should be quantified and mechanism should be elucidated to further understand the effect of polyphenol intake. In addition, investigating the intake of different classes of polyphenol and the association with the adiposity indices should also be considered.

## Figures and Tables

**Table 1:** Sociodemographic characteristics of subjects by sex [presented as number (%)]

Characteristics	Men (*n* = 88)	Women (*n* = 139)	Total (*n* = 227)
Age (years)			
40–49	32 (36.4)	51 (36.7)	83 (36.6)
50–59	56 (63.6)	88 (63.3)	144 (63.4)
Ethnics			
Malay	36 (40.9)	54 (38.8)	90 (39.6)
Chinese	34 (38.6)	50 (36.0)	84 (37.0)
Indian	18 (20.5)	35 (25.2)	53 (23.3)
Educational level			
No formal education	1 (1.1)	11 (7.9)	12 (5.3)
Primary school	21 (23.9)	49 (35.3)	70 (30.8)
Secondary school	59 (67.0)	74 (53.2)	133 (58.6)
Higher education institute	7 (8.0)	5 (3.6)	12 (5.3)
Occupation			
Not working	13 (14.8)	96 (69.1)	109 (48.0)
Pensioner	6 (6.8)	1 (0.7)	7 (3.1)
Pensioner but still working	0 (0)	1 (0.7)	1 (0.4)
Working	69 (78.4)	41 (29.5)	110 (48.5)
Household income			
< RM1,500	15 (17.0)	44 (31.7)	59 (26.0)
RM1,501–RM3,500	70 (79.5)	92 (66.2)	162 (71.4)
RM3,501–RM5,500	3 (3.4)	3 (2.2)	6 (2.6)
Physical activity			
Low	102 (44.9)	24 (27.3)	78 (56.1)
Moderate	98 (43.2)	41 (46.6)	57 (41.0)
High	27 (11.9)	23 (26.1)	4 (2.9)

**Table 2:** Mean for anthropometric profile of the subjects by gender [presented as mean (SD)] (*n* = 227)

Parameters	Men (*n* = 88)	Women (*n* = 139)	*P*-value
Weight (kg)	71.0 (13.2)	66.6 (14.4)	0.019*
Height (cm)	164.8 (5.3)	153.2 (6.0)	< 0.001*
Body fat (%)	27.8 (4.5)	36.7 (6.4)	< 0.001*
NC (cm)	38.4 (3.2)	35.0 (3.3)	< 0.001*
WC (cm)	92.7 (11.2)	92.0 (13.8)	0.755
BMI (kg/m^2^)	26.1 (4.5)	28.4 (6.2)	0.010*

* significant at *P* < 0.010 with Mann Whitney-U test,

NC = neck circumference, WC = waist circumference, BMI = body mass index

**Table 3:** Number and percentage of subjects according to adiposity indices categories by gender [presented as *n* (%)]

Characteristics	Indicator	*n*	Men (*n* = 88)	Women (*n* = 139)
BMI (kg/m^2^)				
Underweight	< 18.5	5	3 (3.4)	2 (1.4)
Normal	18.5–24.9	73	32 (36.4)	41 (29.5)
Overweight	25.0–29.9	86	36 (40.9)	50 (36.0)
Obese	≥ 30	63	17 (19.3)	46 (33.1)
NC (cm)				
Normal	M: < 38; W: < 35	94	26 (29.5)	68 (47.5)
High	M: ≥ 38; W: ≥ 35	133	62 (72.7)	71 (52.5)
WC (cm)				
Normal	M: < 90; W: < 80	68	42 (47.7)	26 (18.7)
High	M: ≥ 90; W: ≥ 80	159	46 (52.3)	113 (81.3)
Body fat (%)				
Normal	M: < 25; W: < 35	75	22 (25.0)	53 (38.1)
High	M: ≥ 25; W: ≥ 35	152	66 (75.0)	86 (61.9)

**Table 4:** Total polyphenol intake, contributions of different food groups to polyphenol intakes and main food sources

Food groups	Mean polyphenol intake (mg/day) [presented as mean (SD)]	Main food contributor (% contribution to polyphenol intake in the food group)
Total foods	1815 (672)	Coffee (20), chocolate milk (10), red beans (7), coffee without milk (7), tea (5)
Fruits	221.8 (162.8)	Orange (33), banana (14), apple (13), watermelon (9)
Vegetables	298.2 (117.5)	Chinese mustard greens (17), onions (12), green beans (8), *kailan* (7)
Cereals and nuts	215.4 (408.1)	Red beans (61), mung beans (19), tofu (7), pistachio nuts (6)
Juice	31.7 (71.2)	Orange (40), lemon (25), apple (18)
Caffeinated beverages	855.8 (477.8)	Coffee (43), chocolate milk (21), coffee without milk (15), tea (11)
Soy	35.0 (57.7)	Soy milk (100)
Chocolate	0.3 (2.3)	Milk chocolate (100)
Alcoholic beverages	0.1 (1.2)	Red wine (87), white wine (13)
Others	156.3 (171.7)	Cinnamon (39), turmeric (23), lemongrass (12), black pepper (6)

**Table 5:** The association between percentile intake of polyphenol and adiposity indices [presented as *n* (%)]

Adiposity indexes	Indicator	*n*	Percentile intake of polyphenol (mg/day)					*χ*^2^	*P*-value

			< 25th (< 1333)	25th–50th (1334–1697)	50th–75th (1698–2164)	> 75th (> 2165)	*P*-value		
BMI							0.745	5.95	-
Underweight	< 18.5	5	1 (20.0)	2 (40.0)	1 (20.0)	1 (20.0)			
Normal	18.5–24.9	73	17 (23.3)	19 (26.0)	17 (23.3)	20 (27.4)			
Overweight	25.0–29.9	86	19 (22.1)	18 (20.9)	23 (26.7)	26 (30.2)			
Obese	≥ 30	63	19 (30.2)	18 (28.6)	16 (25.4)	10 (15.9)			
Neck circumference (cm)									
Normal	M: < 38; W: < 35	94	23 (24.5)	15 (16.0)	27 (28.7)	29 (30.9)	0.040*	8.30	0.032**
High	M: ≥ 38; W: ≥ 35	133	33 (24.8)	42 (31.6)	30 (22.6)	28 (21.1)			
Waist circumference (cm)									
Normal	M: < 80; W: < 90	68	11 (16.2)	15 (22.1)	17 (25.0)	25 (36.8)	0.038*	8.45	0.214
High	M: ≥ 80; W: ≥ 90	159	45 (28.3)	42 (26.4)	40 (25.2)	32 (20.1)			
Body fat percentage									
Normal	M: < 25; W: < 35	75	13 (17.3)	15 (20.0)	26 (34.7)	21 (28.0)	0.045*	8.06	0.067
High	M: ≥ 25; W: ≥ 35	152	43 (28.3)	42 (27.6)	31 (20.4)	36 (23.7)			

Notes:

* significant at *P* < 0.050 by *X^2^* test

** significant at *P* < 0.050 by binomial logistic regression after adjustment of age, income level, caloric intake, physical activity level
